# Five task clusters that enable efficient and effective digitization of biological collections

**DOI:** 10.3897/zookeys.209.3135

**Published:** 2012-07-20

**Authors:** Gil Nelson, Deborah Paul, Gregory Riccardi, Austin R. Mast

**Affiliations:** 1 Institute for Digital Information, Florida State University, Tallahassee, FL 32306-2100, United States; 2 Department of Biological Science, Florida State University, Tallahassee, FL 32306-4295, United States

**Keywords:** Biological specimen collections, paleontological specimen collections, biodiversity informatics, workflow, digitization, curation, imaging, task cluster, iDigBio, ADBC

## Abstract

This paper describes and illustrates five major clusters of related tasks (herein referred to as *task clusters*) that are common to efficient and effective practices in the digitization of biological specimen data and media. Examples of these clusters come from the observation of diverse digitization processes. The staff of iDigBio (The U.S. National Science Foundation’s National Resource for Advancing Digitization of Biological Collections) visited active biological and paleontological collections digitization programs for the purpose of documenting and assessing current digitization practices and tools. These observations identified five task clusters that comprise the digitization process leading up to data publication: (1) pre-digitization curation and staging, (2) specimen image capture, (3) specimen image processing, (4) electronic data capture, and (5) georeferencing locality descriptions. While not all institutions are completing each of these task clusters for each specimen, these clusters describe a composite picture of digitization of biological and paleontological specimens across the programs that were observed. We describe these clusters, three workflow patterns that dominate the implemention of these clusters, and offer a set of workflow recommendations for digitization programs.

## Introduction

This paper presents an analysis and characterization of digitization practices that will help organizations produce and improve effective practices for the digitization of their biological and paleontological collections. The focus is on *digitization workflow*, the sequence of tasks that are performed in order to create digital information that characterizes individual specimens. These tasks typically include photography of specimens and labels, image processing, capture of label information as text, and locality georeferencing. The presentation of workflow characteristics in this paper provides the framework for analyzing the effectiveness and efficiency of workflows and for the development of new effective workflows. It should be noted that the workflows we observed represent a major departure from a historical practice of pulling a single specimen, creating a comprehensive database record, including researching localities, georeferences, collectors, taxon names, nomenclature, and other related details, then moving on to the next specimen (Humphrey and Clausen 1977). This slow data capture process provides an important contrast to the efficient data capture processes examined in this study. It should be further noted that the generalizations we draw here are based on our observations at a select number of institutions and may not encompass the universe of possible digitization workflows. For example, for new specimens, there is a clear trend toward collectors entering data into a database while in the field and this topic is not within the scope of this paper.

We use the term ‘digitize’ to represent the capture and recording of information about a specimen or collection. Specimens typically include labels, accession books, and field notes that have typed or handwritten information about the collection event (e.g. collector’s name, date, locality) and the specimen itself (e.g. scientific name and identifying number). Digitization of label information includes capturing the text as characters, dividing the text into specific properties, and storing this information in a database. Digitization may also include capturing digital images and other media. References to media objects are added to the database records.

The collections community has recognized that digitization processes need to be made more efficient to meet pressing scientific and societal needs (a topic broadly reviewed by Chapman 2005a), a notion supported by such initiatives as GBIF (http://www.gbif.org ), iDigBio (http://www.idigbio.org ) and the Thematic Collections Networks funded by the National Science Foundation’s Advancing Digitization of Biological Collections (ADBC) program (http://www.nsf.gov/pubs/2011/nsf11567/nsf11567.htm), Atlas of Living Australia (http://www.ala.org.au/ ), ViBRANT (http://vbrant.eu/ ), and VertNet (http://www.vertnet.org ). However, little has been published that characterizes modern existing and effective digitization workflows for a broad range of collections (e.g. plant, insect, vertebrate, fossil, microscope slides). We believe such characterizations are an early step in the process of building a common framework for sharing efficiencies across biological and paleontological research collections.

(URLs provided for first mention only. Please see Appendix 2 for URLs of software and websites.)

## Method

This study used the qualitative, *grounded theory research methodology* (Glaser and Strauss 1967, Charmaz 2006) as a general conceptual framework for guiding data collection and analysis. Grounded theory is an inductive social science research method that begins with data collection and leads to qualified conclusions (theories) about those data. The method relies on several techniques useful to our study including simultaneous data collecting and analysis, constructing categories from the data rather than from hypotheses, using a constant comparative method during data collection and analysis, advancing theoretical conclusions during the period of data collection, and sampling aimed at theory construction rather than population representativeness. In the case reported here, categorized concepts from our visits and interviews provided the basis for constructing a modular representation of digitization that we found helpful in describing and elucidating clusters of associated tasks. Data collection included a combination of onsite interviews and observations, analysis of written policies, protocols, and procedures, and the use of multiple observers.

Authors Nelson and Paul, from iDigBio, the U.S. National Science Foundation’s National Resource for ADBC, made onsite visits to 28 programs in 10 museums and academic institutions for the purpose of documenting digitization workflow components and protocols and assessing productivity (Table 1). Workflows were documented photographically, through field notes, and from collected protocol documents provided by visited institutions. Staff members across administrative levels were interviewed, and workflows were carefully observed where possible, either through demonstrations or during real-time data and image capture. Those interviewed included institutional level administrators, biodiversity informatics managers, collections managers, taxonomists and systematists intimately familiar with digitization of specific organismal groups, workflow coordinators, and data entry and imaging technicians. Institutions selected for visitation varied on institution size, collection size, number of ongoing digitization projects, organismal group(s) being digitized, and longevity with digitization activities.

**Table 1.** Summary List of Collections Visited.

**Institution**	**Collections/Programs Visited**	**Collection Size** ^‡^	**Database Software**	**Database Platform**
Yale Peabody Museum (YPM)	**Entomology** ^†^	>1000000	KE EMu	Proprietary
	Invertebrate Zoology	3000000		
	**Invertebrate Paleontology**^†^	350000 lots		
	Vascular Plants	350000		
	Global Plants Initiative			
	Connecticut Plants Survey			
Harvard Museum of Compartive Zoology (MCZ)	**MCZ, Entomology (Lepidoptera**) ^†^	several hundred thousand	MCZbase (Arctos)	Oracle
	MCZ, Entomology (Hymenoptera - Formicidae)	1 million pinned Formicidae		
Harvard University Herbaria (HUH)	**HUH, Global Plants Initiative (GPI**) ^†^	> 5 million	Specify 6, custom	MySQL
	HUH, California Plants			
American Museum of Natural History (AMNH)	Division of Invertebrate Zoology	> 24000000	Planetary Biodiversity Inventory (PBI) for Plant Bugs custom database	MySQL
American Museum of Natural History (AMNH)	Ornithology	> 1000000	KE EMu Microsoft Access	Proprietary
New York Botanical Garden (NYBG)	**Global Plants Initiative (GPI**) ^†^	> 7000000	KE EMu	Proprietary
	**Bryophytes and Lichens (LBCC) TCN**^†^			
	**Tri-trophic (TTD) TCN**^†^			
	Barnaby Legume Monographs			
	**Intermountain Flora**^†^			
	Caribbean Project (ledgers & notebooks)			
	Amazon Project			
	Kohlmeyer Marine Fungus Collection			
University of Kansas (KU)	Biodiversity Institute, **Entomology Collection**^†^	> 4.8 million pinned	Specify 6	MySQL
Botanical Research Institute of Texas (BRIT)	**Apiary Project**^†^ software demo (into ATRIUM database)	> 1000000	Apiary	MySQL
Valdosta State University Herbarium (VSC)	**Vascular Plants**^†^	> 60,000	Specify 6	MySQL
	**Bryophytes**^†^			
Tall Timbers Research Station (TTRS)	**Vascular Plants**^†^	11,000	custom database	MySQL Microsoft Access
	**Lepidoptera**^†^	1200		
	**Ornithology**^†^	4000		
	**Mammalian**^†^	1000		
Robert K. Godfrey Herbarium (FSU)	**Vascular Plants**^†^	> 200,000	custom database	MySQL

† indicates where observers saw the actual digitization process in action.<br/> ‡ number of specimens (unless otherwise stated).

Each site we visited received a questionnaire prior to our visit that examined several categories of digitization tasks that we wished to observe (see Appendix 1). We asked that they use the questionnaire as a guide to prepare for the types of questions we would be asking. The questionnaire was divided into several sections and focused on digitization workflows and tasks. Some institutions completed the questionnaire.

## Task clusters

In the digitization workflows we observed, protocols for the digitization of biological and paleontological specimens were typically divided into clusters of related tasks. The order in which these task clusters were accomplished was based on a combination of staff availability, equipment, space, facilities, institutional goals, and the type of collection being digitized. Hence, though there was a general pattern to the components included within a particular task cluster, the order of accomplishment of the clusters and the tasks within each cluster varied by institution.

These five task clusters were important components of digitization, but not all were essential to meeting the digitization goals of every organization or of every specimen for every organization. These clusters are presented here in a common order of operation:

• pre-digitization curation and staging,

• specimen image capture,

• specimen image processing,

• electronic data capture, and

• georeferencing specimen data.

It should be noted that quality control and data cleaning tasks were integral to each of these task clusters (a topic reviewed by Chapman 2005b, 2005c, Morris 2005, Harpham 2006). Some institutions included a post-digitization quality control step during which data were internally compared for obvious inconsistencies or anomalies, such as discrepancies between the series of a collector’s numbers and the collection dates, data incongruities between local records and duplicates at other institutions, and collection localities outside of a collector’s expected geographic range (a topic reviewed by Morris 2005). This could be considered a sixth task cluster, but we chose to consider it an important part of each of the five task clusters.

## Observed workflow components

### Pre-digitization specimen curation and staging

Curation and staging typically constituted the first step in the digitization workflow, and often had benefits that extended beyond the immediate needs of the digitization program. This step was usually viewed as essential to efficient digitization. Collections managers also reported that it provided a stimulus for attending to needed or neglected curatorial tasks, including opportunities to do the following:

• inspect for and repair specimen damage and evaluate collection health,

• re-pin or remount specimens and replenish or replace preservatives in containers,

• treat specimens for pests,

• attach a unique identifier (most often a 1- or 2-D barcode) to a specimen, container, or cabinet,

• discover important but previously unknown, lost, or dislocated holdings (e.g. those owned by other institutions or the federal government),

• update nomenclature and taxonomic interpretation,

• reorganize the contents of cabinets, cases, trays, and containers, especially when these are the units of digitization,

• vet type specimens, and

• select exemplars for digitization, when that approach is appropriate.

The last five activities in this list may require the greatest knowledge of the organismal group of any during digitization. Many institutions use students, interns, dependable volunteers, or other full- or part-time technicians to accomplish the other pre-digitization curatorial tasks on this list, including the selection of exemplars for digitizing. However, some institutions also reported success with allowing technicians to take on more responsibility for at least some of the last 5 tasks in the above list (Munstermann and Gall 2010).

In addition, as collections data become more generally available online, updating nomenclature and taxonomic interpretations and vetting type specimens can occur after the publication of data and images on the internet, providing an opportunity for off-site experts to comment on the specimens. The latter approach will avoid what can become a bottleneck in the digitization workflow caused by the limited availability of in-house taxonomic experts or well-trained technicians.

Although the application of specimen barcodes is treated here as part of pre-digitization curation, this placement in the digitization workflow is not universal. Some institutions applied barcodes at or just prior to the time of image or data capture, depending on the customized order of operations. In all cases where barcodes were used, they were applied prior to image capture to allow for the barcode value to be seen in the image, and prior to data capture to ensure that the physical specimen identifier is accurately included in the electronic data record.

Barcodes were used for two primary purposes. For individual specimens, barcodes were affixed or pinned to the single specimen or inserted into a wet container that held a single specimen. For specimen groups, such as taxon trays, wet containers, or a collection of specimens from a single collecting event, barcodes were sometimes affixed to or inserted into the enclosing container. In most instances, when a container was barcoded, the number of specimens within the container was recorded, but individual specimens within a common container and not segregated by separate vials were neither barcoded nor otherwise individually identified. When individual vials containing single specimens were aggregated into larger jars, a replica of the label for the containing jar was sometimes inserted into each vial. In a few cases, the container was barcoded as were the individual specimens within that container (e.g. with Lepidoptera). In this latter case, the specimens were digitized individually, with both the individual specimen and container barcodes recorded in the database.

Linear, one-dimensional barcodes are relatively large and are used in cases where sufficient space is available, for example on vascular plant specimens, bryophyte and lichen packets, and other dry, flat specimens. A smaller version of this type of barcode, printed the size of a standard insect label, was also used in entomology collections. Space is an important constraint in barcode selection.

One-dimensional barcodes used for insect collections had two advantages. They mimicked the other labels in size, thus conserving space between specimens, and, if positioned near the bottom of the pin, were easily viewed and hand scanned without removal.

Two-dimensional barcodes were also used, especially for small specimens. They were preferred by some entomology collections because they could be included on an insect pin with the coded end clearly visible and easily scanned.

### Specimen image capture

Determining what to image varied by institution and collection type. Most herbaria imaged entire specimen sheets. Close-up images of particular morphological features (e.g. fruit, flower, or leaf detail) were also sometimes captured. Certain entomological (e.g. ants, butterflies), paleontological, and ornithological collections captured several images of the same specimen with various views (e.g. dorsal, ventral, lateral, hinge, head-on, etc.).

Image acquisition and storage formats also varied by institution (a topic discussed by Morris and Macklin 2006). Many institutions used the Joint Photographic Experts Group (http://www.jpeg.org/committee.html ) (jpeg or jpg) file format for distribution on the internet. Some institutions preferred camera raw formats for archiving images as these formats retain all data originally recorded when the image was made. Others preferred the well-documented and widely used Tagged Image File Format (http://partners.adobe.com/public/developer/tiff/index.html ) (tiff or tif), which retains all of the original image data and most of the Exchangeable Image File Format (EXIF) data (a topic reviewed by Häuser et al. 2005b). Some manufacturers, notably Nikon and Canon, store images in a proprietary raw format that is easily read by manufacturer-produced software, but usually requires software plug-ins to be manipulated by other image editing applications (e.g. Adobe Systems Inc. Photoshop (http://www.adobe.com/products/photoshop.html ) and Lightroom (http://www.adobe.com/products/photoshop-lightroom.html )). It should be noted that capturing and preserving high quality specimen label images offers opportunities to take advantages of future improvements in image analysis (La Salle et al. 2009), optical character recognition (Haston et al. 2012), natural language processing, handwriting analysis, and data-mining technologies.

Manufacturer-controlled raw formats are not openly documented and are subject to change without public notice. Hence, in 2004, Adobe, Inc. developed the publicly documented digital negative format (dng) as well as a freely accessible software application that converts many proprietary raw formats to digital negatives with little or no data loss (http://www.adobe.com/digitalimag/pdfs/dng_primer.pdf ). A few camera manufacturers (e.g. Hasselblad, Leica, Pentax, Ricoh, Samsung) have adopted the digital negative format as the native output for some of their cameras.

From our observations, imaging requires significant specimen handling with attendant opportunities for damage. Hence, most institutions are careful in personnel selection and produce detailed written imaging protocols. However, once an imaging station is installed and properly configured, image acquisition does not appear to be technically challenging and in most institutions we observed is one of the most efficient and productive steps in the digitization process.

Large insect collections sometimes imaged only one label from a single collecting event and applied those data to all specimens associated with that event. Few entomological collections we observed imaged all specimens.

Whereas some institutions imaged only specimens or specimen labels, others included ancillary materials such as collection ledgers (Harpham 2006). Institutions that digitize ledgers typically associate specimen records with the ledger page images that contained additional information about those specimens (see discussion in Australian Museum 2011). Several institutions, especially those with mature digitization programs, expressed the desire to reference external digital objects, such as monographs, published papers, field notebooks, and gray literature to specimen images and records. It is projected that linking such material to specimen records will increasingly become an important enhancement to current specimen digitization protocols.

Imaging station components varied by institution, organism being imaged, and intended use of the resulting images. Most common was a single-lens reflex digital camera fitted with a standard or macro lens and connected to manufacturer or third-party camera control software. A typical station included:

• camera and lens, microscope (for a related discussion, see Buffington et al. 2005), or scanner (HerbScan (see JSTOR PLANTS Handbook http://www.snsb.info/SNSBInfoOpenWiki/attach/Attachments/JSTOR-Plants-Handbook.pdf ) or a custom-designed replica), SatScan (Blagoderov et al. 2010), GigaPan (Bertone and Deans 2010),

• cable connecting camera to computer,

• camera control software (third party or camera manufacturer produced),

• image processing software (most common are Canon Digital Photo Professional (http://www.canon.com ), Nikon Capture NX2 (http://www.nikonusa.com ), Photoshop, and Lightroom), image stacking equipment and software, for example Helicon Focus (http://www.heliconsoft.com/heliconfocus.html ) or Auto-Montage (http://www.syncroscopy.com/syncroscopy/automontage.asp ) (for a related discussion of Auto-Montage, see Antweb (2010)),

• remote shutter release (wireless or tethered),

• copy stand and/or specimen holder,

• studio lighting, flash units, or light/diffuser box (e.g. MK Digital’s Photo EBox Plus (http://www.mkdigitaldirect.com/products/lighting-systems/mk-photo-ebox-plus-1419.html )),

• scale bar,

• color standard,

• stamp to mark that a sheet, jar, tray, or folder had been imaged, and

• associated instruments (pinning blocks, forceps, latex gloves, etc.).

The most common brand of camera in use across collections was a Canon DSLR equipped with a medium-length macro lens, although Nikon DSLR cameras were also sometimes used. Megapixel ratings generally ranged from about 17 to 21.5, but were sometimes lower or higher, depending upon the expected use of the images.

It is instructive to note that generally, the larger the megapixel rating, the better the quality of the resulting images. Hence, images to be used for morphological study were usually captured at megapixel ratings of 17 and above. Macro lenses in the range of 50–60 mm were common, but a few institutions used macro lenses in the range of 100–105 mm, which allowed for close focusing and performed well for smaller objects, such as small birds and mammals. Collections requiring macro images of very small specimens usually used a Leica microscope equipped with a Canon, Nikon, or Leica camera.

To control for image quality, some institutions located the imaging station in a darkened or minimally lit windowless room. This prevented strong extraneous light, like that from a window, from contaminating or overpowering studio lighting or producing visible shadows on the resulting images. Light control was also sometimes accomplished by draping diffuser material across studio lights. A more elegant solution utilized a diffuser box with internal lighting that can be closed prior to image capture. Preferred for this was the MK Photo-eBox Plus Digital Lighting System, originally designed for photographing jewelry, coins, and collectibles. The box is slightly larger than a standard herbarium sheet, rests on a copy stand, includes halogen, fluorescent, and LED lighting, and is equipped with an oval port on the upper surface that allows an unobstructed camera view of the specimen. Herbaria using this system usually place the color bar and scale at the top of the sheet to preserve the aspect ratio of the resulting image, thus obviating the need for image cropping and reducing the number of steps required for image processing. Although the requirement to open and close the doors of the light box seemingly slowed the imaging rate, time lost was likely recaptured from a reduction in time spent on post-imaging batch cropping and light level adjustments.

HerbScan is the imaging system used for scanning type specimens for the Global Plants Initiative (GPI) project (http://gpi.myspecies.info/ ). GPI specifications require that specimens be scanned at 600 ppi resolution, beyond the capacity of most DSLR cameras when used for whole sheet images of herbarium specimens. HerbScan uses a flatbed scanner (Epson Expression Model 10000XL, Graphic Arts, USB2 and Firewire interfaces) and a platform that raises the specimen sheet to the face of the inverted scanner. Scanning requires 4-6 minutes per scan for a maximum effective rate of about ten images per hour. Because the specimen sheet is pressed against the rigid glass face of the scanner, the acceptable depth of the specimen sheet is limited to about 1.5 cm, hence some specimens are too bulky for this equipment.

Keeping up with what has and has not been imaged can be daunting, especially in large collections. Many collections that we observed used the presence of a barcode or a stamp to indicate whether a particular specimen had been imaged and/or digitized. Herbaria often stamped the sheet or folder at the time of imaging to provide a visible demarcation. Some institutions also used a written or electronic tracking system to track digitization in an orderly fashion. Electronic tracking was usually accomplished within the database management system being used for data storage. For many institutions, deciding what to digitize was based on such criteria as responding to special projects, processing loan requests, emphasizing centers of interest, a desire to focus on unique or important parts of the collection, or other priorities. In such instances, an electronic tracking system ensured that specimens were not overlooked.

Maintaining an organized tracking system for actively growing collections is especially dependent on effective protocol. Some institutions included digitization within the accessioning workflow, ensuring that all newly acquired specimens, especially those to be inserted into parts of the collection that had been previously digitized, were handled at the time of specimen acquisition.

Workflow requirements for imaging varied by institution, but generally followed a similar pattern:

• pre-imaging equipment configuration and initialization,

• procuring/organizing the next batch of specimens for imaging,

• acquiring the image, and

• moving specimens to the next station or re-inserting them into the collection.

Pre-imaging equipment configuration and initialization was generally a one-time task accomplished at the beginning of an imaging session. It involved:

• connecting or ensuring the connection of computer to camera,

• starting external studio lighting, or checking, adjusting, and testing flash units and power supplies,

• starting camera control and image acquisition software,

• starting the camera,

• setting camera aperture, shutter speed, and focus point (or loading these attributes from a previously configured settings file),

• adjusting camera height,

• changing or attaching lenses, and

• loading ancillary image management/processing software.

**Figure 1.**
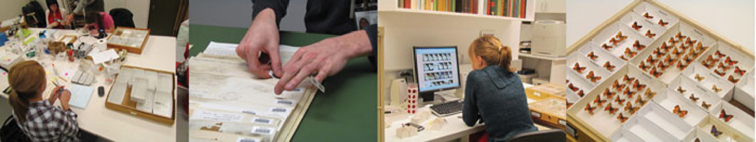
Pre-digitization specimen curation and staging. Preparing barcodes and imaging labels, affixing barcodes, updating taxonomy. L to R: University of Kansas – Entomology, New York Botanical Garden and Yale Peabody Museum.

**Figure 2.**
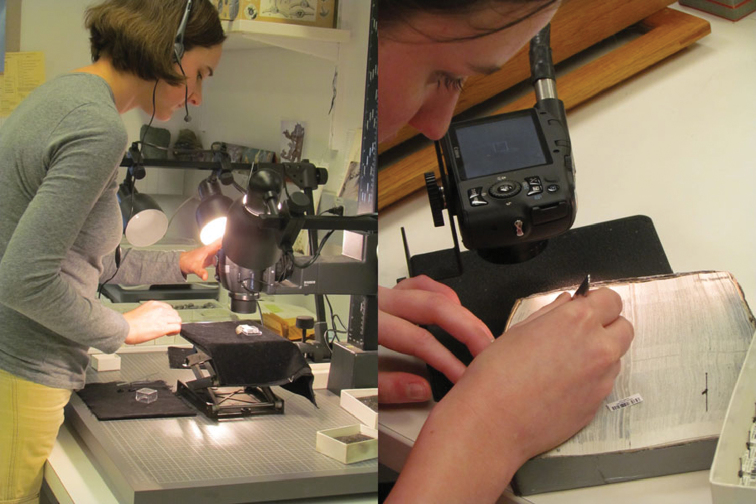
Specimen image capture. Fossil specimen imaging, specimen label imaging. Two very different imaging set-ups. Yale Peabody Museum, University of Kansas - Entomology.

In some institutions, especially those where all specimens are similarly sized (e.g. herbaria), camera settings and equipment mountings were usually not changed from session to session and required only a spot check prior to commencing a new imaging session. With collections of variously sized organisms (e.g. paleontological, ornithological, Lepidopteran), camera distance to subject was frequently adjusted, lighting re-arranged, camera settings altered, and custom or specialized specimen holders repositioned. In some instances, grouping like-size specimens alleviated the need for continuous camera adjustment and increased workflow efficiency. In these situations, the potential increase in imaging error due to increased demands for technician judgment were effectively offset by a higher level of detail in written protocols, elevated attention to specialized training, and diligent monitoring during the early phases of a new technician’s tenure. Institutions that imaged only labels that required only moderate resolution sometimes dispensed with much of the equipment listed above in favor of a small digital camera and less elaborate copy stand that afforded more mobility (Figure 2).

Procuring and organizing the next batch of specimens for imaging was sometimes facilitated by ensuring proximity of the specimens to the imaging station. Institutions used mobile carts or cabinets to transport specimens from the pre-digitization curation or data entry areas to a location in close proximity to the imaging station. Moving specimens from station to station rather than returning them to storage cabinets and re-retrieving them reduced the amount of time devoted to travel and handling. From our observations, workflows that began with image capture, imaged every specimen, and extracted data directly from the image rather than the physical specimen effectively eliminated the need to handle or move specimens beyond the imaging stage, facilitating re-storage immediately following imaging (Figure 6c). To ensure that specimens did not get misplaced and potentially lost within the collection, re-filing specimen drawers, trays, containers, or folders was often reserved for curators or technicians intimately familiar with collection organization. To facilitate the smooth flow of specimens, staging space was often made available at every station where physical specimen handling was required.

Image acquisition focuses on the process of camera operation for image capture. For collections with standard sized specimens (e.g. herbaria), the process involved repeating a rote procedure for each new specimen. Even for such collections, however, the technician was required to pay close attention to quality by periodically examining images to ensure that:

• lighting, exposure, and focus remained constant,

• file naming progressed according to plan,

• exposure was correct,

• focus remained sharp,

• images lacked imperfections such as blemishes or streaking,

• files were not corrupted, and

• barcodes or identifiers were in place and readable.

**Figure 3.**
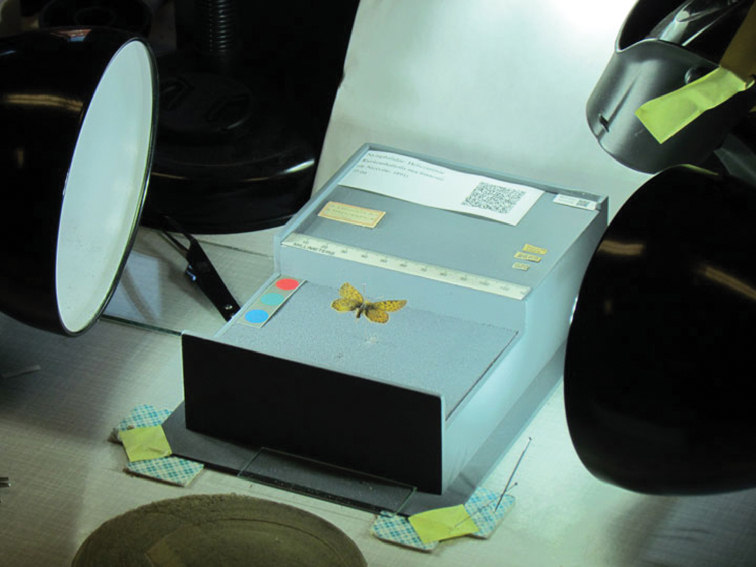
Custom specimen holder. Museum of Compartive Zoology (MCZ) Rhopalocera (Lepidoptera) Rapid Digitization Project.

For wet collections, exemplar specimens were usually removed from the container before imaging. One successful technique we observed for imaging fish, reptiles, amphibians, and other organisms with a reflective epidermis submerged them in a shallow, ethanol-filled container, allowed the ripples to settle, and acquired the image through the ethanol. This method increased detail by reducing reflectance and increasing contrast. Coating fossil specimens with a thin layer of alcohol also increases contrast and provides for a sharper image (Paul Selden, personal communication, 2012).

Protocols and workflows for efficiently imaging insects—with the possible exceptions of bees, ants, and butterflies—are under development and continue to pose special challenges. In nearly every case where we observed butterflies being imaged, specimens were removed from the pinning substrate, labels were carefully removed and placed on a custom-designed holder with the labels and barcodes (or other identifier) clearly visible in the resulting image. One institution (Museum of Comparative Zoology) designed and constructed a custom specimen holder (Figure 3) with sufficient space to include all labels and the specimen in a single image (Morris et al. 2010). Other institutions rested the specimen on a parallel pair of taut monofilament lines and recorded two views (dorsal and ventral), each with one or more labels visible (see Häuser et al. 2005a). Some institutions combined the dorsal and ventral views side-by-side in a single composite image using image management software such as ImageMagick (http://www.imagemagick.org/ ).

Imaging productivity varied by collection. For herbaria, rates per imaging station ranged from as few as 10 sheets per hour using a single HerbScan, to 75–120 sheets per hour using a camera (average rate slightly less than 100 sheets per hour). Imaging rates for insects are not well documented and their derivation is sometimes confounded by the inclusion of data entry and image acquisition in a single, linear workflow that makes it difficult to segregate strictly imaging tasks from data entry. For example, the imaging step might include removing the label from the pin, taking the photo, and putting the label(s) back on the specimen pin.

### Specimen image processing

Image processing involves all tasks performed on an image or group of images following image capture. Nine tasks are addressed here, reflecting common practices:

• quality control,

• barcode capture,

• file conversion,

• image cropping,

• color balance or light level adjustments,

• image stacking,

• redaction,

• file transfer, and

• optical character recognition (OCR).

Some institutions include one or more of these nine tasks (e.g. barcode capture, OCR) at other stages of the digitization process, as noted in the discussion below.

**Figure 4.**
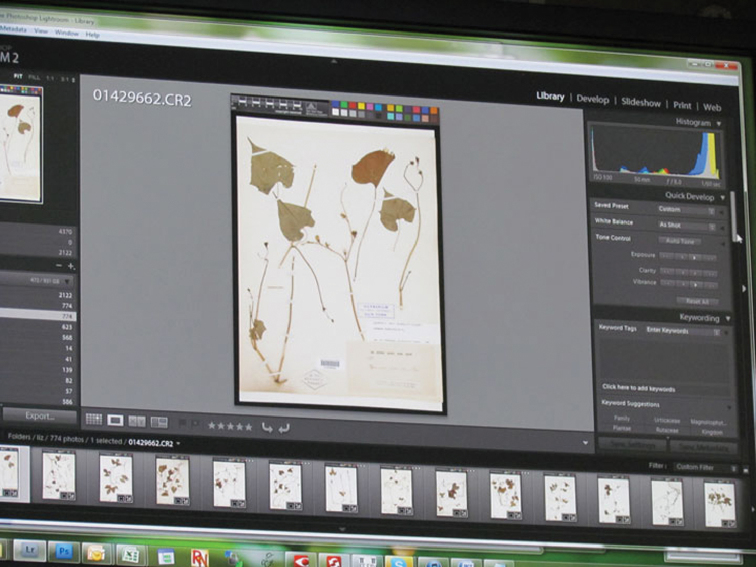
Specimen image processing. Using Adobe Photoshop Lightroom software to process images. New York Botanical Garden.

Quality control was usually effected by selecting and examining sample images at regular intervals. In some institutions, all images were visually scanned for obvious deficiencies before individual images were selected for more thorough review. Selected images were evaluated for correct focus and exposure, blemishes, scan lines, mismatches between file names and barcode values (in situations where these are expected to match), and other obvious signs of imperfections or errors. Imperfections in camera images usually related to incorrect focus or exposure. Institutions using HerbScan, especially as part of the GPI, followed a more elaborate and rigorous process (not detailed here) that included converting images to high contrast in Photoshop and running scripts that track pixilation and banding, and that expose scanner-produced flaws such as minute streaks and lines caused by wear and tear on scanner parts. The standard for GPI images, coupled with mechanical parameters of the scanners, demanded these enhanced quality control procedures (http://www.snsb.info/SNSBInfoOpenWiki/attach/Attachments/JSTOR-Plants-Handbook.pdf ).

Barcode values were captured in several ways and for several purposes. Many institutions preferred specimen image file names to match corresponding specimen barcode values. Hence, the image file for a specimen with barcode value XXX123456, might be named XXX123456.tif, where XXX is replaced by the institution code. This worked well for cases in which each specimen was represented by a single image, but less effectively for cases in which a specimen might be represented by multiple images. In these latter cases, multiple image files of the same specimen often used an appended value, such as XXX123456A, XXX123456B, and so forth. Although matching the image filename to the specimen’s barcode value is not a requirement, it is a common practice that helped ensure that all image files for a specific collection were uniquely named.

Based on our observations, collections that chose to use barcode values as filenames generally used one of several options. Most high-end DSLR cameras allow for customized file naming and auto-incremented file numbering, features sometimes used in herbaria. When these features were used simultaneously, the camera was configured to produce file names that matched the barcode value. This increased efficiency when specimens were arranged and imaged in sequential barcode order, but was cumbersome and inefficient when specimens were arranged in random barcode order. It also led to file naming errors when one or more specimens were unexpectedly mis-ordered. A second practice used a barcode scanner to read the barcode into the file name field or the image EXIF data as the file was imaged or saved. A third strategy used Optical Character Recognition (OCR) software to scan the image file for a barcode value and rename the file to the barcode value detected. The benefits of the latter approach included reduction of potential naming errors and greater efficiency due to reduced camera manipulation.

However, OCR software sometimes failed at detecting barcodes within images due to image quality or other issues, resulting in files not being appropriately renamed. According to our observations, barcode extraction failure rates on bryophyte packets ranged from 0.2–3%, based on tests with ABBYY Finereader Corporate edition (http://finereader.abbyy.com/corporate/) at the herbarium of Valdosta State University, where barcodes were carefully affixed in precise horizontal or vertical orientation. A fourth approach used custom-designed software to intercept the filename generated by the camera, simultaneously creating an associated record in the database for later data entry from the image. Image filenames were unique for the collection, and the image files were usually stored in a repository and linked to database records through a software interface.

A two-part strategy we observed that addressed file naming issues used a hand-held scanner to scan the barcode value into the image EXIF via Canon Digital Professional software. Subsequent processing extracted the image’s barcode value using ZXing (Zebra Crossing, http://code.google.com/p/zxing/ ), compared the value to the image’s EXIF data, and created a database record containing the image filename and barcode value. This allowed database records to be created by software without regard to the image’s filename. The key point of this process is that camera-generated filenames can be stored verbatim in a database if software is responsible for associating image files with specimen records (Morris and Macklin 2006).

Conversion involves converting camera raw images to a preferred archival or display format. In some instances, conversion is avoided by setting the camera to record images in the preferred final archive format, usually as a tagged image file (tif).

Cropping is used to trim excess image data in order to achieve an acceptable aspect ratio or to reduce unnecessary borders surrounding the specimen. Where cropping was utilized, it was accomplished in large batches that did not require monitoring once set into motion. However, cropping was not universal.

In general practice, it is considered unwise to use photo manipulation software to alter color balance, saturation, sharpness, or other image features (Cromey 2010). Doing so runs the risk of creating an image that does not faithfully represent the source specimen. Based on our observations, adjustment of light levels is an exception to this rule. Herbarium specimens, in particular, sometimes benefitted from an automatic levels adjustment. An auto levels adjustment essentially sets the white and black points in the image and spreads the available tones between these two extremes. Using an auto-levels adjustment worked best when the image contained a color bar that included true black and white reference points. This gave a better representation of the tonal values between the extremes, and usually resulted in a more lifelike image without distorting color or other attributes. Since all herbarium specimens in a specific photographic session were presumably recorded with equal illumination, consistent camera settings, and the same lens, all images made within that session benefited equally from a batched adjustment. The same was not always true for colorful subjects, such as birds or butterflies, which often responded to auto levels adjustments in a way that distorted the resulting images, often rendering them more colorful and brighter than the original.

Specimens with significant depth, such as fossils, some insects, birds, mammals, and even some herbarium sheets, make it difficult to achieve sharp focus throughout the depth of field. Institutions used one of several stacking software packages to rectify this problem. Focus stacking (http://en.wikipedia.org/wiki/Focus_stacking ) involved recording several images of a stationary specimen at varying depths of field, processing them through a stacking algorithm that essentially merged the several layers into a single image while preserving properly focused pixels in each layer. The result was a sharply focused image throughout the specimen’s depth. Software packages in common use included proprietary Auto-Montage (see discussion in Antweb 2010) and Helicon Focus. No-cost software included CombineZ (http://www.hadleyweb.pwp.blueyonder.co.uk/CZP/Installation.htm ). Stacking worked best with cameras that supported a live view of the specimen in conjunction with camera control software that allowed precise focus control targeted to small percentage regions of the specimen.

### Electronic data capture

Electronic data capture involves extracting label data and entering those data into an electronic database. Depending on protocol, data capture can occur before, after, or simultaneous with image capture. For collections we observed in which all or nearly all specimens were to be imaged, entering data from specimen images reduced specimen handling and potential damage, eliminated multiple trips to storage locations, and allowed technicians to digitally enlarge labels for better readability. For collections that did not image specimens, or imaged only exemplars, data entry was usually the second step in the digitization sequence (Figure 6a).

**Figure 5.**
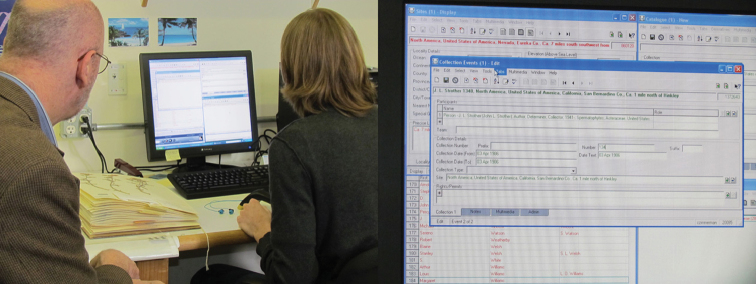
Electronic data capture. Entering data straight from the specimen label into the database. New York Botanical Garden.

**Figure 6.**
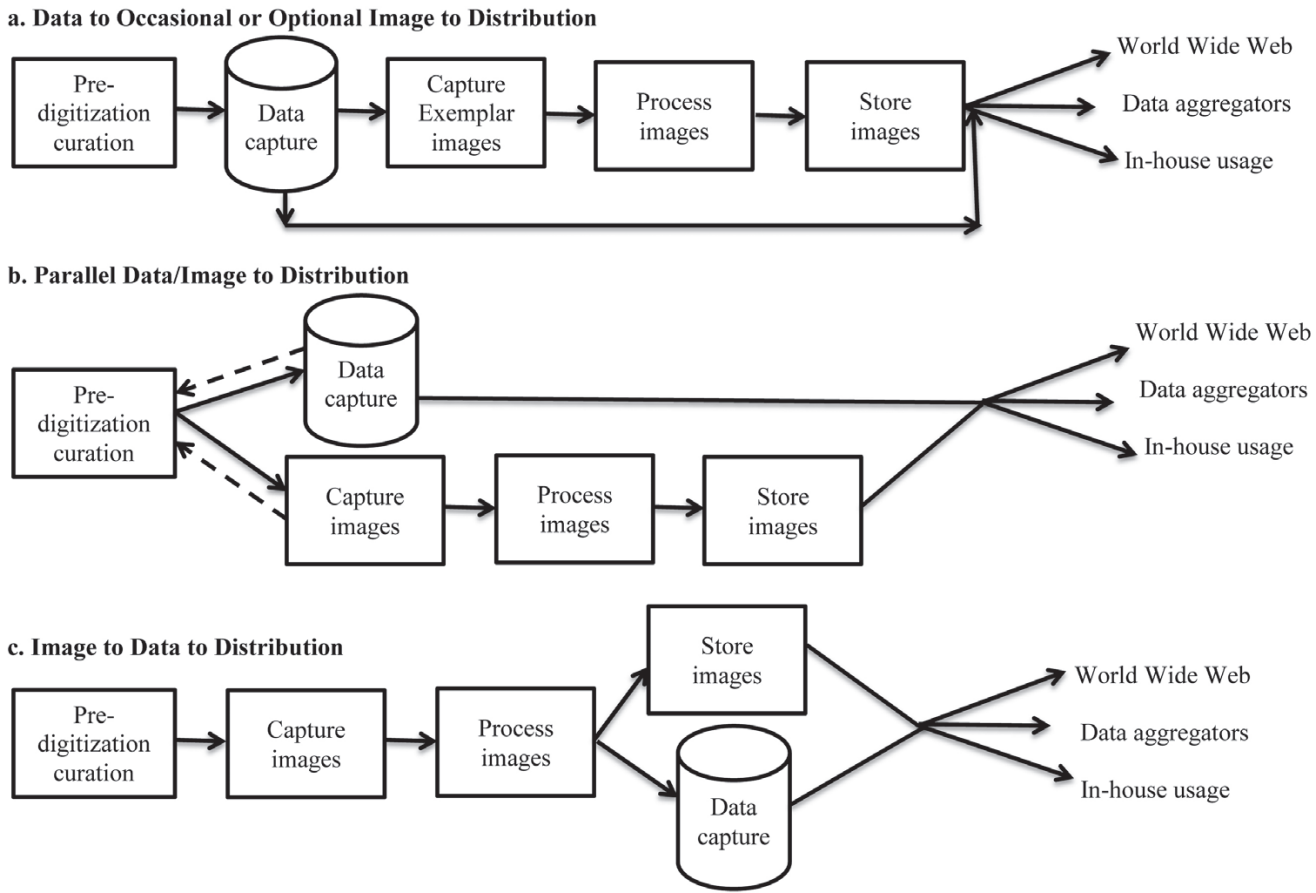
Dominant Digitization Workflows Observed.

Several methods were used for data capture, the most common being keystroke entry, sometimes with the support of related technologies such as OCR or voice recognition. Efficiently designed software interfaces that allowed user customization were important and increased the efficiency of data entry by eliminating duplicative or unnecessary keystrokes and arranging icons in convenient positions or in logical tab orders (see related discussion in Morris 2005). We noted that in almost all cases, the database software used in a given collection was not used out-of-the-box. Often, software was customized or custom-designed user interfaces were built by biodiversity informatics managers.

Advances in voice recognition technology are evident in computer, tablet, and smart phone applications. Nevertheless, we saw only a single use of this technology, and this only for capturing a limited set of data, but we note that some institutions are experimenting with this technology. IBM ViaVoice (now produced by Nuance Communications, Inc. (http://www.nuance.com/ )), Microsoft Voice Recognition (a standard component of the Microsoft Windows® operating system), and Dragon Naturally Speaking (http://www.nuance.com/for-business/by-product/dragon/dragon-for-the-pc/dragon-professional/index.htm ) are three software packages being used or tested. We note that programmers at the Botanical Research Institute of Texas (BRIT) are testing the Application Programming Interface that is packaged with the Microsoft Windows® operating system.We believe that voice recognition shows great potential for data capture and that the comparatively small cost for appropriate commercial products will be offset by greater workflow efficiencies. Most modern operating systems include built-in voice recognition capabilities of various qualities that should be tested using a high quality microphone. From our experience, the potential drawback to this technology is that substantial training to particular voices is often required for the software to perform adequately, which may limit its use where several data entry technicians are involved or when the rate of technician turnover is high. In addition, we noted from our interviews that simultaneous data entry by several technicians in close proximity might lead to distortion and interference, or be distracting to workers.

Optical character recognition (OCR) was also being used or considered by several institutions. Two of the most effective uses we observed included the Apiary Project (http://www.apiaryproject.org/ ) at BRIT and the Symbiota Software Project (http://symbiota.org/tiki/tiki-index.php ) at Arizona State University. Each of these interfaces simultaneously displays a specimen image, an OCR-rendered version of label data extracted from the image, and a collection of database fields into which data can be transferred. Apiary allows users to demarcate OCR regions of interest within the image and highlight OCR-generated text that can be transferred to associated data fields by mouse click. Symbiota provides for moving data to fields manually, but additionally includes functionality for searching the databases of the Consortium of North American Byrophyte Herbaria (http://symbiota.org/bryophytes/ ) and Consortium of North American Lichen Herbaria (http://symbiota.org/nalichens/ ) for previously digitized duplicates from which data can be imported.

Other institutions routinely process all images through OCR and store the OCR-generated output in text files, or import it into a field within the database for subsequent editing, data cleaning, and searching. Popular OCR software packages included Tesseract (http://code.google.com/p/tesseract-ocr/ ), OCRopus (http://code.google.com/p/ocropus/ ), and JOCR (GOCR) (http://jocr.sourceforge.net/ ), all of which are open source, and the proprietary ABBYY Finereader corporate version (http://www.abbyy.com/ ) and Adobe Acrobat Professional version (http://www.adobe.com/products/acrobatpro.html ), both of which can batch process large numbers of images. There is significant interest in natural language processing (NLP), which is designed to parse OCR text into fields, as well as intelligent character recognition (ICR) or handwriting analysis, but effective systems for using these technologies to extract data from biological specimens were not observed.

In some instances data entry is accomplished by electronic import from spreadsheets or other delimited lists. Some software interfaces, e.g. Specify (http://specifysoftware.org/ ) (via Workbench), Brahms (http://herbaria.plants.ox.ac.uk/bol/ ) (via Rapid Data Entry http://herbaria.plants.ox.ac.uk/bol/BRAHMS/Documentation ), and KE EMu (http://www.kesoftware.com/ ) provide this capability. Issues to resolve when importing legacy or external data include data quality, mapping imported data fields to those in the preferred database, dealing with imported fields that do not have database correlates, and time required for post-import data cleanup. In many cases, importing and transforming legacy data can be efficiently managed, resulting in large dataset acquisitions for relatively small investment in time, especially when compared to keystroking.

### Georeferencing

Georeferencing is the process of transforming textual descriptions of geographical data into a pair of X, Y coordinates, with an accompanying estimation of precision. Precision is usually denoted by one of several methods, including a bounding polygon, a point and its associated radius of uncertainty, or designation of the extent of the known area in which the point occurs, such as a county, park, township, range, or section (Chapman and Wieczorek 2006). Best practices suggest that each georeferenced point also include notation of the point’s datum, geographical coordinate system, and georeference remarks that explain how the point, polygon, and estimate of precision were derived (Chapman and Wieczorek 2006). Coordinate pairs that do not include notation of the underlying datum upon which the point is based may include uncertainties up to about 3.5 km (Wieczorek et al. 2004).

Based on our observations, the process of georeferencing biological and paleontological specimens was typically ancillary to and discontinuous with the digitization workflow. Although digitization workflows often captured locality information from specimen or collecting event labels, these data—especially legacy data—generally did not contain geographical coordinates and most institutions chose not to georeference these data at the time of data entry. In the case of more recently collected specimens on which latitude and longitude values were included on the label, the values were typically captured at the collecting event or specimen record level at the time of data entry. It is clear from our observations that the community consensus for legacy specimens is for bulk georeferencing of unique localities as a separate step in the digitization workflow (Chapman and Wieczorek 2006).

We observed three georeferencing methodologies in use where coordinate values were not present on the specimen. Geolocate (desktop and web-based interfaces, and web services; http://www.museum.tulane.edu/geolocate/ ) and Biogeomancer (web-based; http://bg.berkeley.edu/latest/ ) are software applications designed to assist in assigning latitude/longitude coordinates to textually described localities. Both of these applications convert locality descriptions into coordinate pairs based on statements of state, county, orthogonal direction, distance, and place names of geographical features. Both also provide protocols for uploading datasets for processing and bulk georeferencing similar localities. Each returns a map of the estimated location of each described locality, including a point-radius estimate of precision. Map interfaces allow technicians to manipulate and refine the georeferenced locations of these points before recording a final determination of the point’s coordinates. Technician manipulation was required for points to be reliable. Both Geolocate and Biogeomancer are free to use. The third method we observed was based on the use of standard and customized map layers in conjunction with GIS software (such as ArcMap http://webhelp.esri.com/arcgisdesktop/9.2/index.cfm?TopicName=An_overview_of_ArcMap ) and paper maps to pinpoint locations. For best results, all of these systems rely on a technician’s knowledge of the region in which a collection is made, facility with desktop GIS or online mapping software, general understanding of maps and mapping, and ability to recognize habitat signatures on aerial photographs.

### Dominant digitization workflows observed

Based on our observations, three workflows dominated digitization programs in the institutions we visited (Figure 6). The three presented here are not intended to represent a comprehensive collection of workflows. Here we call them by their characterizing patterns: *data to occasional or optional image to distribution*, *parallel data/image to distribution*, and *image to data to distribution*. All patterns begin with pre-digitization curation and terminate with distributing data directly to the World Wide Web, to data aggregators, and/or to internal users. In all three, specimen data are stored in database records that include references to associated images or other media. Images are stored in a computer file system and are not embedded in the database. We have not measured the throughput of these patterns in a controlled experiment.

It is worth noting that the capture of specimen data from ledgers without reference to the specimens has been a dominant digitization workflow for many decades and represents the method by which the majority of existing vertebrate collections data were digitized (Humphrey and Clausen 1977). With one exception, this method was absent from the workflow patterns we observed in this study, likely due to the transition in recent years to digitizing directly from specimens.

We note that Tann and Flemons (2008) and Granzow-de la Cerda and Beach (2010) provide examples of how one might measure a data capture workflow for a given collection type. These might serve as models for setting up comparisons of workflows across or within collection types.

The *data to occasional or optional image to distribution* pattern fits those institutions in which few or no specimens are imaged. Data capture follows curation and may include decisions about which specimens to submit for imaging. Rarely, imaging of exemplars is simultaneous with data entry of those exemplars.

The *parallel data/image to distribution* pattern includes both data and image capture but treats them as independent and simultaneous rather than as sequential steps. This pattern is likely the most labor intensive of the three, especially when it requires specimen handling at two stages of the workflow, with attendant need for multiple trips to storage locations and increased opportunities for specimen damage. This pattern is made more efficient when data capture proceeds from bulk data sources (ledgers, cards), which requires specimen handling only during image aquisition.

The *image to data to distribution* pattern fits institutions that image all specimens (e.g. most herbaria) and captures data from these images. It reduces specimen handling and with it the likelihood of specimen damage, increases efficiency by eliminating the need for return trips to storage locations, and offers the capacity to incorporate Optical Character Recognition and similar technologies within the data capture workflow.

## Recommendations

Based on our observations, interviews, discussions, and readings, we offer the following recommendations for establishing and improving biological and paleontogical collections digitization programs.

1. With planning, the pre-digitization curation step is an opportunity for the goals of specimen digitization and collection curation to be merged into an efficient workflow. Curation tasks that cannot be efficiently addressed in the workflow can be identified so that adequate resources can be assigned to them in the future (Sumpter 1991).

2. Biodiversity informatics managers and other digitization personnel should look for bottlenecks in digitization workflows and seek ways to make them more efficient (Tann and Flemons 2008; Granzow-de la Cerda and Beach 2010). We recognize that much work remains for devising and disseminating strategies for evaluating and analyzing existing workflows, encouraging the application of automation, and exploring the relevance of industrial process control to workflow design.

3. There should be clear institutional policies guiding which specimens to expose to public access, including policies governing whether to redact or not redact locality data for sensitive species (Canhos et al. 2004) and ensuring that permission is obtained for privately controlled donations and collections from federal installations. We note, for example that funds from NSF’s Advancing Digitization of Biological Collections are not permitted to be used in the digitization of federally owned specimens (National Science Foundation 2011).

4. Barcodes should be used only as identifiers; encoded barcode data should not incorporate taxonomic or related information that might change with time.

5. Where possible, the aspect ratio of specimen to camera should be synchronized to eliminate the need for image cropping.

6. Image processing should not include color balancing or other adjustments that result in images inaccurately reflecting actual specimens (Cromey 2010).

7. A color bar and scale should be visible in all images (Taylor 2005).

8. Protocols for periodic quality control should be established for all stages in the digitization workflow to ensure data accuracy and the production of high quality digital images (Chapman 2005a).

9. For institutions in which imaging is paramount, acquiring images of labels prior to data entry reduces specimen handling by allowing for data extraction from images rather than from specimens.

10. Attention to the digitization of gray and published literature related to specimen data is an important consideration and should be accomplished whenever possible (cf. Australian Museum 2011).

11. Georeferencing should be treated as an essential part of digitization protocols (Canhos et al. 2004, Chapman and Wieczorek 2005, Morris 2000).

12. Quality control should be integral to all steps in the digitization workflow, including post-digitization review and targeted testing should be designed to expose data inconsistencies or suspected anomalies (Morris 2005).

13. Detailed written protocols should guide every step of the digitization workflow, be uniquely designed for a given institution, and be amended regularly to reflect emerging technologies and improved efficiencies. These protocols should be electronically stored in a common folder that allows technicians to insert comments and suggestions to be reviewed and potentially adopted by biodiversity informatics managers.

14. Selection of data entry and imaging technicians should be guided by employability skill sets strongly associated with success in digitization tasks, with particular attention to potential technicians’ attention to detail, orientation to increased efficiency, and commitment to high productivity.

15. Institution-wide digitization tasks should be periodically evaluated for overall progress, organizational collaboration and cooperation, and compatibility with new and emerging technology, with plans to use results of the evaluation to implement improvements (Kalms 2012).

16. Digitization workflows should be coordinated by a designated biodiversity informatics manager with IT experience, preferably from a biological sciences and collections background, to bridge the potential knowledge gap between collections managers and information technology professionals (Kalms 2012).

17. Biodiversity informatics managers should construct a frequently asked questions document that outlines common problems and offers instructions about how to address these problems, whom to contact with questions about specific categories of problems, and guidelines for which types of problems should be elevated to a higher administrative level.

18. Institutions should utilize a digitization workflow strategy that captures problems, remedies, lessons learned, and technician input for use in improving digitization protocols, and remain open to investigating possible changes in current practice (Kalms 2012).

19. Determining an appropriate storage format for archived images is an important decision that should precede image capture. Here we recommend capturing images in native camera raw and converting them from camera raw to dng or tif (a topic addressed by Häuser et al. 2005b). Alternatively, images can be natively captured and archived in tif format. Jpg format is not recommended for archived images.

## Appendix 1

Questionnaire. (doi: 10.3897/zookeys.209.3135.app1) File format: Reach Text Format (rtf).

**Explanation note:** Questionnaire used for interviewing staff of visited collections.

## Appendix 2

Web resources. (doi: 10.3897/zookeys.209.3135.app2) File format: Microsoft Word Document (docx).

**Explanation note:** Web resources mentioned in the paper.
